# Body Image: A Bridge Between Depression and Quality of Life in Women With Cancer

**DOI:** 10.1002/pon.70328

**Published:** 2025-11-05

**Authors:** Lavinia Rotella, Matteo Aloi, Elvira Anna Carbone, Renato de Filippis, Daria Quirino, Marianna Rania, Valeria Saladino, Francesca Cuzzocrea, Pasquale De Fazio, Cristina Segura‐Garcia

**Affiliations:** ^1^ Department of Medical and Surgical Sciences University “Magna Graecia” of Catanzaro Catanzaro Italy; ^2^ Department of Clinical and Experimental Medicine University of Messina Messina Italy; ^3^ Outpatient Unit for Clinical Research and Treatment of Eating Disorders University Hospital Renato Dulbecco Catanzaro Italy; ^4^ Department of Health Sciences University “Magna Graecia” of Catanzaro Catanzaro Italy

**Keywords:** body image, breast cancer, cancer, depression, quality of life, self‐concept

## Abstract

**Background:**

Cancer and its treatments often result in visible bodily changes and emotional distress, affecting self‐perception, mood, and overall well‐being. Body image, particularly in women with breast cancer, plays a key role in shaping psychological adjustment and quality of life. However, its potential mediating role between depression and quality of life in cancer populations remains underexplored.

**Aims:**

This cross‐sectional study investigated whether body image statistically mediates the relationship between depression and quality of life in female patients with cancer and whether this differs between patients with breast cancer compared to other cancer diagnoses.

**Methods:**

Overall, 160 women with cancer aged 18–65 were recruited from a single clinical site. Participants completed the Body Image Scale (BIS), the depression subscale of the Hospital Anxiety and Depression Scale (HADS‐D), and the EORTC QLQ‐C30 to assess quality life. U‐Mann Whitney tests were used for group comparisons. Mediation analyses testing two separate models for breast cancer and non‐breast cancer groups were conducted.

**Results:**

In breast cancer group, body image statistically mediated the relationship between depression and quality of life. Depression was associated with greater body image distress, which in turn, predicted lower quality of life. In contrast, among patient with other cancer diagnosis, depression directly impacted quality of life without a significant mediating effect from body image.

**Conclusions:**

These findings underscore the unique psychological burden of breast cancer and highlight the importance of addressing body image in psychological interventions to improve the quality of life in this population.

## 1 Introduction

Body image is the subjective representation individuals hold about their own body, independent of how it appears [1, 2]. It is a multifaceted construct comprising affective, cognitive, perceptual, and behavioural components [3].

Negative body image can adversely affect both psychological and physical health, self‐worth, affective state, social interactions, and general functioning.

In individuals facing chronic illness such as cancer, body image is particularly vulnerable. The physical consequences of oncological conditions and their treatments—surgery, chemotherapy, radiotherapy, hormonal changes—often result in visible bodily changes, including hair loss, weight fluctuations, scarring, and early menopause, among others, all of which can threaten self‐esteem and bodily identity [4, 5].

These changes are frequently experienced as traumatic, acting as constant reminders of illness and diminishing feeling of femininity ad attractiveness [6]. Cancer represents a double trauma: a physical assault on the body and a psychological rupture in the person's sense of self. The emotional distress accompanying diagnosis and treatment may range from vulnerability and sadness to clinical depression and anxiety, undermining patients' capacity to engage in daily life and relationship [7].

Furthermore, menopause introduced by cancer treatments can have vasomotor, genitourinary, cardiovascular, and skeletal repercussions, all of which are tied to reduced sexual functioning and identity, further impacting psychological well‐being [8, 9].

These physical and psychological factors converge to shape body image experiences in cancer patients [10, 11, 12]. When body perception and self‐esteem are compromised, the risk of depression increases, and overall quality of life declines [13, 14]. Evidence shows that depressive symptoms and altered body image may reinforce each other, forming a vicious cycle of emotional and perceptual distress [15]. The presence of fatigue, pain, and changes in appearance adds further complexity, potentially intensifying depressive symptoms and interfering with coping mechanism [16]. Restoring a positive body image in this context is essential to improving self‐esteem and enhancing quality of life. Acceptance of bodily changes—rather than striving for pre‐illness appearance—has been identified as a key psychological adjustment strategy [17]. Taken together, the literature suggests that body image, depression, and quality of life are deeply interrelated in oncology patients [18, 19, 20]. However, breast cancer may present a unique psychological profile, given its profound impact on female identity, femininity, and sexuality [21]. Yet, the precise role of body image as a potential mediator between depression and quality of life in breast cancer patients versus those with other cancer types remains underexplored [22, 23, 24].

From a theoretical perspective, body image can be explained in Cash's cognitive‐behavioral model [25] that describes body image as a result of interactions among perceptual, cognitive, affective, and behavioural processes. Adopting this model, negative appearance‐related schemas and maladaptive self‐evaluative standards can worsen emotional distress, particularly when the body undergoes noticeable changes such as those caused by cancer and its treatments. In oncology populations, these cognitive‐affective processes can contribute to depressive symptoms and quality of life detriments through self‐discrepancy and reduced self‐acceptance. The inclusion of this model allows for a more comprehensive explanation of how body image functions as a potential psychological bridge between depression and well‐being in women with cancer.

The current research aims to investigate the association between body image, depression, and quality of life in women with cancer, focussing on differences between breast cancer and other cancer diagnoses. We hypothesized that higher depression levels would be associated with poorer body image and lower quality of life. Furthermore, we expected body image to mediate the relationship between depression and quality of life in women with breast cancer, but not in patients with other cancers—highlighting the distinctive psychological burden in breast cancer contexts.

## 2 Materials and Methods

### 2.1 Participants

In this cross‐sectional research, women receiving care at the Day Hospital of the Medical Oncology Unit of the *XXXX (anonymized)* were invited to participate. Recruitment took place between February and June 2025.

Eligible participants were women between 18 and 65 years of age with a diagnosed oncological condition, capable of independently completing self‐administered questionnaires, and able to give informed consent.

Exclusion criteria: patients with medical conditions or undergoing treatments that could interfere with their ability to understand and complete the questionnaires; patients with a current or past history of eating disorders or body dysmorphic disorder. Further records with missing values for any of the variable of interest were excluded from the analysis in order to ensure that all the observation included in the study contained fully observed data necessary for the mediation model.

**FIGURE 1:**
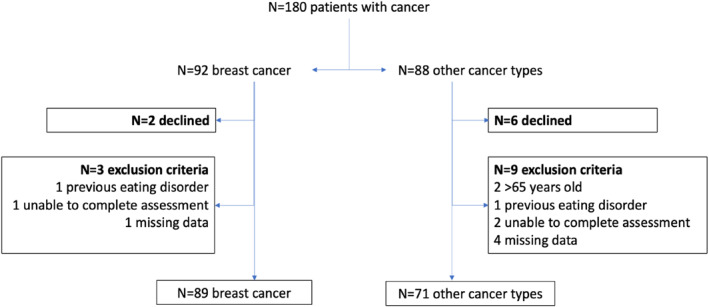
Participants allocation.

In total, 180 female patients were screened for eligibility. Of these, 20 were excluded ‐ 8 declined participation and 12 failed to meet the inclusion criteria (i.e., 2 were older than 65 years old, 2 had a life‐time diagnosis of an eating disorder, 3 were unable to complete the assessment on their own, and 5 had missing data). This resulted in a final sample of 160 patients who met the eligibility requirements and agreed to take part in the study (Figure 1). Most patients were diagnosed with breast cancer (*n* = 89, 55.6%). The remaining 71 patients presented with other cancer types, including gynaecological cancers such as ovarian or uterine cancer (*n* = 26, 16.3%), colorectal cancer (*n* = 14, 8.8%), pancreatic cancer (*n* = 10, 6.3%), haematological malignancies (*n* = 7, 4.4%), stomach or oesophageal cancer (*n* = 6, 3.8%), bladder cancer (*n* = 4, 2.5%), and lung cancer (*n* = 4, 2.5%). A higher proportion of patients without breast cancer reported a family history of psychiatric disorders (*n* = 6, 8.5%).

In line with previous studies, the number of participants was determined in advance according to the “n:q criterion”, which considers the ratio between the sample size (*n*) and the number of model parameters (*q*) [26]. To achieve adequate statistical power, a ratio of 10 participants per parameter was set. Given a total of six parameters for each model (see Statistical Analysis section), this corresponded to a minimum required sample size of 60 participants per model tested. All participants received information regarding the purpose of the investigation, the voluntary participation, the anonymity of own data and the possibility to withdraw from the research at any point of the procedure. Prior to any assessment, every participant signed a written informed consent declaring their understanding and agreement to take part in the study. The Territorial Ethical Committee of Regione Calabria approved the current study. The research was carried out in compliance with the ethical principles of the Declaration of Helsinki [27].

### 2.2 Assessment

Patients were interviewed during a follow‐up visit. A custom questionnaire was created for collection of socio‐demographic data: sex, age, civil status, employment, and educational attainment. Data on disease duration, treatment type (e.g., surgery, chemotherapy, radiotherapy), presence of metastases, and other relevant clinical variables were retrieved from patient medical records.

The subsequent self‐report questionnaires were individually administered.

- *Body Image Scale (BIS)*: It evaluates perceptions of body image and potential distress in oncology patients [28]. It is unidimensional 10‐items scale Likert type addressing behavioural (e.g., avoiding mirrors of people), emotional (e.g., “feeling feminine”, “feeling attractive”), and cognitive (e.g., satisfaction with body or surgical scars) components. The present study used the Italian version [29]. The total score range from 0 to 30, with higher values reflecting greater distress. McDonald's *ω* was 0.916.
- *Hospital Anxiety and Depression Scale (HADS)*: The HADS is a commonly used self‐report questionnaire developed by [30] to assess anxiety and depression in non‐psychiatric medical patients. It is commonly used in oncology and other medical settings due to its exclusion of somatic symptoms (e.g., headache, insomnia, weight loss) that might stem from the illness or treatment. The scale is made up of two subscales—anxiety (HADS‐A) and depression (HADS‐D)—each with 7 items. Scores range from 0 to 21 for each subscale. Scores of > 11, 8–10 and < 7 respectively indicate probable, borderline, and no anxiety/depression. The HADS has proven to be a valid tool for identifying negative affect in patients with cancer due to its specificity for medical contexts, good psychometric, brevity, rapid administration, and high compliance. In the current research, we used the Italian validation [31]. McDonald's *ω* was 0.772.
- *EORTC QLQ‐C30 (version 3)*: The European Organization for Research and Treatment of Cancer (EORTC) develop this questionnaire to measure quality of life in oncology populations [32]. Version 3 evolved from earlier ones starting in 1978 and consist of 30 items. It includes multi‐item and single‐item scales: five functional domains (emotional, social, role, physical, cognitive), three symptom domains (nausea/vomiting, pain, fatigue), and one global health/QoL scale. In addition, six single‐item measures evaluate symptoms and issues typically associated with cancer and its treatment, including appetite loss, dyspnea, constipation, insomnia, diarrhoea, financial difficulties. Patients are asked to answer all items with no “right” or “wrong” answers. The first 28 questions are using a 4‐point response format (1 = Not at all, 2 = A little, 3 = Quite a bit, 4 = Very much), and the last two use a 7‐point Likert scale. All scales and single‐item measures are scored from 0 to 100. Higher score on functional and global health/QoL scale indicate better quality of life, while higher symptom scores indicate greater symptom burden. Scoring involves computing the mean of the item in each scale, followed by linear transformation to standardize scores on a 0‐100 scale. The QLQ‐C30 is the most used QoL tool for cancer patients across Europe, and it is also used in the Americas and worldwide. Its psychometric properties have been extensively validated. In this study, the Italian validated version of the scale was administered [33]. McDonald's *ω* was 0.885.

### 2.3 Statistical Analysis

The Social Sciences Statistical Package, Version 26.0 (SPSS Inc., Chicago, IL, USA) was used for data analysis. To determine the appropriate statistical tests, normality of variables as well as group equivalence was first tested using the Shapiro‐Wilk test. Sociodemographic variables met the normality assumption; thus, group differences were assessed using Student's *t*‐test and the chi‐squared test.

Since length of illness, depression, quality of life, and body image variables were not normally distributed, the non‐parametric Mann‐Whitney *U* test was applied. For outcomes that reached statistical significance, effect size *r* was calculated.

In line with the research question, the strength of the association among body image, depression, and quality of life, as well as the mediational pathway, was examined. To verify the linearity assumption among the dependent, independent, and mediator variables, we first inspected scatterplots and a correlation matrix.

JASP software was employed to perform mediation analyses were conducted using (JASP Team, 2024). Two separate models were tested: one for patients with breast cancer and another for patients with other types of cancer. In both models, the depression subscale of the HADS was treated as the independent variable, quality of life as the dependent variable, and body image as the mediator.

The statistical significance of the mediating and indirect effects was assessed using a bootstrap procedure (with 5000 percentile‐based confidence intervals) and the maximum likelihood robust (MLR) estimator, following the approach outlined by [34].

## 3 Results

The socio‐demographic and clinical features of patients with breast cancer versus other cancer types are shown in Table 1. No statistically significant differences were found between the two groups in age (*t* = 1.425, *p* = 0.35), education (*χ* ^2^ = 4.201, *p* = 0.24), employment (*χ* ^2^ = 0.262, *p* = 0.99), marital status (*χ* ^2^ = 7.422, *p* = 0.12). The proportion of patients reporting no current psychiatric disorder was slightly higher in the breast cancer group; however, the difference was not statistically significant (*χ* ^2^ = 5.186, *p* = 0.08). Finally, no differences were evident in relation to the length of illness.

**TABLE 1:** Socio‐demographic and clinical features of the sample.

	Breast cancer	Other types	Statistics	*p*
Age (years)^#^	52.1 (7.8)	53.4 (9.5)	*t* = 1.425	0.35
Educational level				
Primary school	5 (5.7)	5 (7.0)	*X* ^2^ = 4.201	0.24
Middle school	36 (40.4)	19 (26.8)		
High school	35 (39.3)	30 (42.3)		
Bachelor's degree	13 (14.6)	17 (23.9)		
Employment				
Unemployed	16 (18.0)	12 (16.9)	*X* ^2^ = 0.262	0.99
On disability	2 (2.2)	2 (2.8)		
Unpaid work	21 (23.6)	18 (25.3)		
Employed	40 (44.9)	30 (42.2)		
Retired	10 (11.3)	9 (12.8)		
Civil status				
Single	7 (7.9)	12 (16.9)	*X* ^2^ = 7.422	0.12
Married/Cohabiting	73 (82.0)	46 (64.9)		
In a relationship (not cohabiting)	4 (4.5)	5 (7.0)		
Separated/Divorced	1 (1.1)	4 (5.6)		
Widowed	4 (4.5)	4 (5.6)		
Psychiatric disorders				
None	80 (89.9)	58 (81.7)	*X* ^2^ = 5.186	0.08
Anxiety	1 (1.1)	6 (8.4)		
Depression	8 (9.0)	7 (9.9)		
Familiarity for psychiatric disorders				
Yes	0 (0.0)	6 (8.5)	*X* ^2^ = 7.814	**0.01**
No	89 (100.0)	65 (91.5)		
Length of illness (months)°	12.00	9.00	*U* = 2820.00	0.76

*Note:* Values are presented as frequencies (%) unless otherwise specified.

^#^ Data presented as means and (Standard deviation).

° Length of illness data are shown as median. Significant differences in bold character.

Table 2 presents a comparison of body image, depression, and quality of life between patients with breast cancer and those with other cancer types. Overall, the results suggest that the two groups were comparable across the variables of interest.

**TABLE 2:** Difference in body image, depression, and quality of life in breast cancer patients versus patients with other cancer types.

	Breast cancer (*N* = 89)	Other types (*N* = 71)			
	Mean rank	Mean rank	U	Z	*p*
BIS	85.2	74.5	3584.5	1.46	0.14
HADS‐D	84.8	75.2	3539.5	1.31	0.19
QL2	80.0	81.1	3115.0	−0.15	0.88

Abbreviations: BIS: Body image scale; HADS‐D: Hospital Anxiety and Depression Scale—Depression subscale; QL2: Quality of life.

### 3.1 Mediation Analyses

Mediation analyses were conducted to examine whether body image statistically mediates the relationship between depression (HADS‐D) and quality of life (QL2) in two separate patient groups: those with breast cancer and those with other cancer types. Path analyses were performed using the maximum likelihood estimator with robust standard errors. Direct, indirect, and total effects were estimated, and significance was evaluated via bootstrap confidence intervals and delta method as appropriate. As shown in Table 1, no significant differences emerged between the two groups in key sociodemographic or clinical variables (age, education, employment status, marital status, and length of illness); therefore, no covariates were included in the mediation analyses, as these variables were not expected to confound the relationships among depression, body image, and quality of life.

#### 3.1.1 Breast Cancer Patients

**FIGURE 2:**
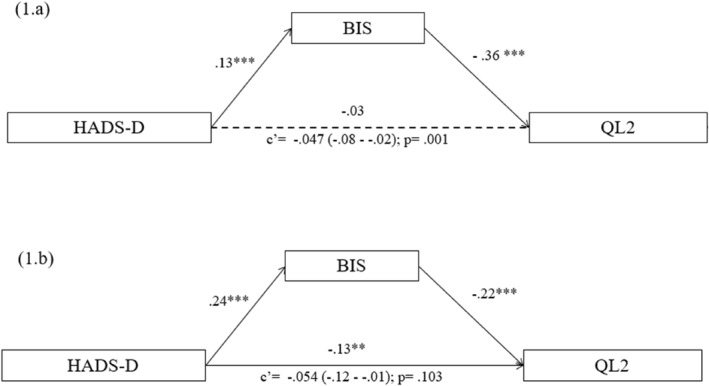
(2.a) path diagram model in patients with breast cancer; (2.b) path diagram model in patients with other type of cancer.

In the breast cancer group (Figure 2a), the direct effect of depression on quality of life was not statistically significant (*β* = −0.032, *p* = 0.359, CI 95% [‐0.10, 0.03]), suggesting no direct association between depressive symptoms and quality of life in this subgroup. However, the indirect effect through body image was statistically significant (*β* = −0.047, *p* = 0.004, CI 95% [‐0.08, −0.02]), indicating that higher depression levels were associated with lower body image appreciation, which in turn predicted poorer quality of life.

Path coefficients further supported these results: depression significantly predicted body image distress (*β* = 0.130, *p* < 0.001, CI 95% [0.07, 0.19]), and body image was a significant predictor of lower quality of life (*β* = −0.361, *p* = 0.001, CI 95% [‐0.58, −0.14]). These findings highlight body image as a significant mediator of the impact of depression on quality of life in breast cancer patients.

#### 3.1.2 Patients With Other Types of Cancer

In contrast, for patients with other types of cancer (Figure 2b), the direct effect of depression on quality of life was significant (*β* = −0.128, *p* = 0.010, CI 95% [‐0.23, −0.03]), indicating that depressive symptoms were directly associated with lower quality of life. The indirect effect through body image, however, was not statistically significant (*β* = −0.054, *p* = 0.103, CI 95% [‐0.12, 0.01]), suggesting that body image did not mediate the relationship in this group. Regarding the path coefficients, depression was positively associated with body image disturbance (*β* = 0.241, *p* < 0.001, CI 95% [0.17, 0.31]), but body image did not significantly predict quality of life (*β* = −0.223, *p* = 0.093, CI 95% [‐0.48, 0.04]). Thus, no mediating effect of body image was supported in this subsample.

Together, these analyses indicate a differential pattern across groups. Among breast cancer patients, body image plays a key statistical mediating role in the relationship between depression and quality of life. In contrast, among patients with other cancers, depression impacts quality of life primarily through a direct path, with no significant mediating effect of body image. These results highlight the unique psychological dynamics related to body image in breast cancer and encourage the relevance of targeted interventions for body image in this specific population.

## 4 Discussion

The present study examined the interplay between body image, depression, and the quality of life in female oncology population, differentiating between those diagnosed with breast cancer and those with other cancer types. Across both groups, higher levels of depressive symptoms associated with poorer body image and reduced quality of life [35]. Several research have confirmed the negative impact of cancer diagnosis, especially breast cancer, and associated treatments on body image, sexuality, and levels of anxiety and depression levels [16, 36, 37, 38]. These psychosocial difficulties may exacerbate the challenge of coping with cancer, leading to a deterioration in affective and social well‐being [15].

Interpretation of the mediation effects in this study is statistical rather than causal. Although the analyses demonstrate that body image statistically mediates the relationship between depression and quality of life, the cross‐sectional design precludes any causal inference regarding directionality among these variables.

In this framework, body image was found to statistically mediate the relationship between depression and quality of life only in the breast cancer group, as hypothesized. In contrast, for patients with other cancer types, depression had a direct impact on quality of life, and body image did not exert a significant mediating effect. These findings fully supported our initial hypotheses.

Our findings support previous models of mediation that have investigated the role of body image in psychological adjustment to breast cancer. For instance, Amini‐Tehrani and colleagues demonstrated that body image indirectly influenced psychological distress via enacted and internalized stigma, confirming central position of appearance‐based self‐concept in emotional consequence [22]. Similarly, Li demonstrated that body image was associated with depressive symptoms and post‐traumatic growth via reduced social support [15]. While these studies hypothesized body image more as a predictor than a mediator, together they highlight its pivotal role in the emotional and social dimensions of cancer adjustment. Our model, however, operationalizes body image as a mediator through which depressive symptoms affect quality of life in a more general sense, extrapolating existing evidence to a more general understanding of well‐being.

Our results emphasize the unique psychological dynamics associated with breast cancer, where changes in body image appear to play a central role in emotional well‐being and perceived quality of life. This likely reflects the deep connection between physical appearance, femininity, and self‐identity in this population. The breast holds particular symbolic importance in relation to female identity, attractiveness, and sexuality.

Changes caused by the clinical condition and treatment of breast cancer—particularly mastectomy—can result in profound body image disruptions with short‐ and long‐term consequences for emotional health and quality of life. Unlike most other cancers, breast cancer is often associated with surgical amputation, which may generate intense negative emotional responses [39].

Women who underwent breast‐conserving therapy or reconstruction reported a more positive body image, greater sexual satisfaction, improved partner relationship and increased feelings of attractiveness [40] compared with those who underwent mastectomy, who often experienced feelings of inadequacy regarding their femininity [41, 42, 43].

The latter group often experienced difficulty accepting their altered body and reported low quality of life [44].

On the other hand, other type of cancer can also affect body image and psychological health.

However, the depressive symptoms and reduction in quality of life observed in these patients may more often result from treatment‐related symptoms—such as pain, fatigue, lack of energy, irritability, insomnia, appetite loss, or gastrointestinal problems—rather than from appearance‐related concerns. These effects may independently contribute to emotional distress and impaired quality of life, without body image playing a significant mediating role [6].

Additionally, the presence of a BRCA1 or BRCA2 mutation, which indicates a high risk of developing breast cancer, has been linked to greater psychological distress and diminished quality of life, even in the absence of an active cancer diagnosis [45].

### 4.1 Clinical Implications

These findings highlight the need for personalized psycho‐oncological care that directly addresses the specific emotional and cognitive challenges of breast cancer patients. Given that body image statistically mediates depression‐quality of life, therapeutic interventions should directly target appearance‐based assumptions, self‐criticism, and treatment‐induced identity reconstruction.

Evidence‐based interventions such as cognitive‐behavioral therapy (CBT) for body image have been proven to reduce body dissatisfaction and improve emotional adjustment by challenging negative self‐schemas and facilitating cognitive restructuring of appearance‐related beliefs [25, 46, 47]. Acceptance and commitment therapy (ACT) is also able to facilitate patients to develop psychological flexibility and acceptance of body changes and thereby reduce experiential avoidance and improve the quality of life [48, 49, 50]. Compassion‐focused therapy (CFT), as it focuses on self‐compassion and defusing self‐criticism, may also be particularly beneficial for women who suffer from shame, loss of femininity, or altered self‐concept following surgery or treatment [51, 52].

Applying these strategies together in multidisciplinary oncology care may help the patients develop a more loving and compassionate relationship with their bodies, decrease depressive symptoms, and eventually promote long‐term psychological health and quality of life.

### 4.2 Strengths and Limitations

An important strength of the current research is its multidisciplinary approach, which explores the complex interplay between depression, body image, and quality of life across physical, psychological, and social domains. By considering these different dimensions, the research offers a more holistic understanding of the lived experiences of cancer patients—especially those of women with breast cancer—highlighting how these factors intertwine to shape their overall well‐being.

Additionally, the relevance of the topic to the psychological health of cancer patients underscores its potential value in informing and guiding the development of target psychological and therapeutic interventions.

Despite these strengths, several limitations should be acknowledged. First, as stated above, the cross‐sectional study does not permit the establishment of causal relationships between depression, body image, and quality of life. Although the mediation model provides some information about the relationships among these variables, direction suggested (“depression affects body image, which affects quality of life”) has to be interpreted carefully. Longitudinal and experimental study in the future has to be done in order to determine the temporal order and causality of the relationships.

Second, heterogeneity of the comparison group is a methodological weakness. Convenience‐recruited non‐breast cancer patients had a variety of oncological diagnoses (e.g., gynaecological, colorectal, pancreatic, and hematologic cancers), which are likely to differ considerably in their physical, emotional, and psychosocial implications. This heterogeneity could have reduced group comparability and limited the generalizability of our results. More homogeneous and matched samples should thus be employed in future research to further separate diagnosis‐specific psychological processes.

Third, the study was conducted in a single oncology ward of the Italian healthcare system and may limit the external validity of the findings. Cultural and contextual features of the Italian oncology settings—femininity norms, body image, and emotional expression norms—may have influenced participants' experiences and responses. Replication in multicenter trials and cross‐cultural studies is recommended in order to enhance the generalizability of these findings.

Finally, variation across participants in treatment and time since diagnosis could have influenced perceptions of psychological well‐being and body image. Future longitudinal research must investigate how these variables alter across treatment phases and survivorship trajectories to establish intervention‐sensitive periods.

## 5 Conclusion

The association between body image, depression and quality of life in patients with breast cancer is complex and multifactorial. Women with breast cancer have distorted body image, possibly linked to disease‐related bodily changes and the consequences of treatments. Chemotherapy, radiotherapy and surgery that can negatively affect patients' self‐perception and psychological health, contributing to higher level of depression and lower quality of life.

Further research is needed to better identify and characterize and diagnose body image concerns among cancer patients in general, but breast cancer, will improve healthcare approaches aimed at reducing treatment‐related negative impacts in breast cancer survivors. In fact, increased satisfaction with one's body and sense of femininity may act as a protective factor in coping with breast cancer and its associated treatments [53].

In particular, access to targeted psychological and supportive care may enhance patients' mental health and quality of life, providing a more hopeful trajectory for recovery.

## Author Contributions

**Lavinia Rotella:** writing – original draft, writing – review and editing, **Matteo Aloi:** methodology, writing – original draft, writing – review and editing. **Elvira Anna Carbone:** data curation. **Renato de Filippis:** Data curation. **Daria Quirino:** data curation. **Marianna Rania:** writing – review and editing. **Valeria Saladino:** writing – review and editing. **Francesca Cuzzocrea:** writing – review and editing, supervision. **Pasquale De Fazio:** conceptualization, supervision, writing – eview and editing. **Cristina Segura Garcia:** conceptualization, data curation, methodology, supervision, writing – review and editing.

## Funding

The authors received no specific funding for this work.

## Ethics Statement

The study followed all relevant ethical guidelines. All procedures performed were in accordance with the ethical standards of the institutional research committee and with the Helsinki Declaration (or comparable ethical standards).Funding

Declaration of generative AI and AI‐assisted technologies in the writing process.

During the preparation of this work, the author(s) did not employ generative AI or AI‐assisted tools for content creation, data analysis, or writing. All content was produced, reviewed, and edited solely by the author(s), who take full responsibility for the content of the published article.

## Consent

All participants provided informed consent, and the research was conducted according to the best principles of research with human beings.

## Conflicts of Interest

The authors declare no known conflict of interests, including financial interests or personal relationships that could have appeared to influence the work reported in this paper.

## Data Availability

Data supporting the results of this study are available from the corresponding author upon reasonable request.
